# Governing bio-objects: a research agenda

**DOI:** 10.3325/cmj.2012.53.80

**Published:** 2012-02

**Authors:** Janus Hansen, Ingrid Metzler

**Affiliations:** 1 Department of Business and Politics, Copenhagen Business School, Copenhagen, Denmark *jh.dbp@cbs.dk*; 2 Life-Science-Governance Research Platform, University of Vienna, Vienna, Austria *ingrid.metzler@univie.ac.at*; On behalf of the members of Working Group 2 "The Governance of Bio-objects" of the COST Action "Bio-objects and their Boundaries: Governing Matters at the Intersection of Society, Politics and Science", see *http://www.univie.ac.at/bio-objects*

Over the past few decades, we have encountered life in surprising new forms and in unusual spaces. Scientists have been able to derive stem cells from the inner cell masses of early human embryos and to keep these cell lines dividing and growing; mammals have been produced not through the fusion of gametes but with the help of cloning technologies; and mice have been “humanized.” Some of these entities have themselves been transformed and made mobile. Human embryonic stem cell lines derived from an embryo, for instance, have been “coaxed” into forming more specific and dedicated cell types, or they have found their way as bio-repositories. The rising number of such entities or “bio-objects” – as the recently established research network “Bio-objects and their boundaries: Governing matters at the intersection of society, politics, and science,” funded by the COST program, suggests naming them – bears witness to the growing ability of the life sciences to control life, to intervene in it, and sometimes also quite literally to “make” it. The salience of these bio-objects in public spaces – such as institutions entrusted with the ordering of our collective life – in newspaper headlines or internet fora for patient groups, testifies to the hopes that many individual and collective actors in post-industrial societies invest in the life sciences, which are entrusted not only with the task of producing knowledge but also with the task of making our collective life healthier, safer, and more productive.

However, debates and controversies on these innovative entities, as well as on the technologies and practices that help to make and to sustain them, suggest that the process of bio-objectification should not be understood as a one-way street. Such debates include, on one hand, controversies on who or what is amenable to be “objectified” – and how, but also less vociferous debates in which scientists, policy-makers, and other groups of actors discuss how to order these entities, who to entrust with their oversight, and in light of what sort of principles. We discuss this latter aspect in this contribution, which is the third in a series of contributions on “bio-objects” and “bio-objectification” processes (1,2).

We briefly report on the research objectives of a Working Group within this research network that focuses on the “governance of bio-objects.” This group explores and compares how various bio-objects have been governed, seeks to identify commonalities and differences, and seeks to learn how different modes of governing bio-object shape and interfere with bio-objectification processes. Here, we identify some of the dimensions that help us make this comparison in practice. However, before we outline these dimensions, we briefly clarify what kind of social processes and institutions we refer to as “governance of bio-objects.”

## The governance of bio-objects

Over the past three decades, the concept of governance has experienced a rapid rise in use both within and outside of the social sciences. However, its precise meaning remains ambiguous, and contested.

Two broad approaches can be identified: Some scholars use *governance* as a counter-concept to *government*, to designate the emergence of new modes of ordering and coordinating that operate beyond and/or by other means than traditional state authorities, for instance by delegating responsibilities to non-state actors such as companies or civil society organizations. Scholars in this tradition talk about “governance without government” or about shifts from “government to governance,” referring to an increase in network-based coordination of different societal activities. Other scholars use *governance* more generally to refer to various modes of steering or coordinating, independently of whether state-actors are involved or not. Distinctions are made between hierarchies, markets, and networks as the main types of coordination mechanisms; however, the suggestion is that such mechanisms exist next to another. We base our work on the latter, more encompassing approach. This means we apply the concept of governance to all kinds of steering and coordination mechanisms that are brought to bear on bio-objects. Using this more generic understanding of governance, we compare the various modes in which bio-objects are ordered 1) across “polities,” such as different countries or nation states; 2) across different types of bio-objects, such as stem cells or genetically modified organisms; and 3) across time, for example, by exploring temporal developments of governance within a particular fields, or “learning effects” that spill from one field to another. Here we sketch some of the dimensions with which we make this comparison in practice.

## Location of responsibility or agency

The first dimension of comparing different modes of governance relates to the question of who is entrusted with governing and overseeing particular bio-objects. Here, it is customary to distinguish between public and private domains of activity and responsibility, although some governance literature is occupied with the various ways in which this distinction is crossed, blurred, or dissolved in more network-like topographies of responsibility and distributed agency. Hence, in practical terms, this dimension can be considered as a continuum ranging from domains where public bodies are entirely responsible and operate in a conventional, hierarchical fashion through hard law, monitoring, and sanctioning (eg, traditional risk management), to domains that are left entirely to the private sector – such as the self-governance of science through its own bodies of oversight or through entrusting individual citizens or consumers with making their own informed decisions. In general, the location of governance and the allocation of rights and responsibilities tend to follow fairly established patterns. However, bio-objects tend to blur boundaries between such pre-established locations, for instance, by “sitting-in-between” traditions of scientific self-governance and interventions by state-authorities.

## Drivers of governance

The second dimension in the comparison of different modes of governance that requires clarification is whether bio-objects trigger calls for revising existing regulations, institutional frameworks, or established boundaries. Such calls are often made. This does not imply that pre-established frameworks are always modified or eliminated altogether. However, pre-existing modes of governance tend to be modified and adapted. One way to compare these adaptations is to explore who the actors or “drivers” are that foresee the need for regulation, and how that need is framed in terms of responsibilities, rights, and duties. Drivers may include the perceived need to protect public goods, such as the physical safety of human beings and the environment, the moral and ethical dignity of human beings and animals, or the safeguarding of privacy. The drivers of regulation might just as well emerge from civil society organizations concerned with “human dignity” in light of developments in reproductive technologies, but they may also result from the needs of commercial actors to have legal clarification before making investment decisions.

## Purpose of governance

The third, related, dimension for a comparison between different modes of governance relates to the “purpose” of governance. Do actors argue that there is a need to revise regulatory frameworks in order to confine a bio-object to particular space or to impede the “objectification” of particular forms of life? Or is the need for a revision of regulatory mechanisms framed in terms of ensuring the facilitation of bio-objectification processes or enabling smoother transitions from a bio-object as a promissory research object in the laboratory to an approved therapy in the clinic? There are instances in which actors call for regulatory barriers that contain – or disallow – particularly innovative creatures from coming into being. Often such barriers are drawn even before such innovative creatures exist. Bans on the reproductive cloning of human beings are an example of such a restriction or a permanent ban. In practice, however, these two dimensions often go hand in hand. Genetically modified organisms regulation can serve as an example: the regulatory frameworks put in place by the European Commission are meant not just to facilitate the development and implementation of the technology, but also to protect human health and the environment from undesirable side effects (and, it can be argued, to protect the political system against public opposition to the technology). The key question in this dimension pertains to how different purposes of governance are configured in relation to each other – and possibly made the object of social controversy about the goals of governance.

## Temporality of governance

A fourth dimension in our comparison of different modes of governance relates to the temporality of governance. This refers to whether governance is prospective or reactive in relation to bio-objectification. Does governance aim to steer bio-objectification in particular directions (eg, setting research agendas, designating social or medical problems to be solved) or is it mostly reacting to emerging bio-objects, either to control undesirable effects or to search for potential applications of generic technologies?

In some domains and in some polities, it seems that there is a general shift under way from reactive to more prospective or proactive modes of governance. For instance:

• GMO regulation is increasingly about identifying potential hazards to human health and the environment prior to deliberate releases of new GMOs. This raises the “cognitive costs,” as regulators need not only to understand observable phenomena, but also to model potential consequences.

• “Public engagement” activities are increasingly directed at “upstream” activities, ensuring public engagement with research and innovation agendas, rather than simply reacting to “downstream” outcomes.

Key questions in this dimension are thus not only the actual temporal ordering of “innovations” *vis-ŕ-vis* “regulation” (whether regulatory frameworks are constantly behind or whether they actively shape innovations), but also more fundamentally what the “temporal ambitions” of different regulatory tools and frameworks are.

## Extension of the modes of governance

While all processes of bio-objectification are local in some sense, some processes reach wider or are more extensively networked than others. This also pertains to the mechanisms of governance. Hence, fifth, we need to ask at what level processes of governance are located and at what level they (attempt to) exert influence. This reaches from very local practices, pertaining to perhaps just one particular laboratory or biobank organizing its work in a particular manner, over national frames of legislation to international and global regimes of governance, such as international patent laws or international conventions like the Cartagena protocol on biodiversity. The key question to ask in this dimension of comparison pertains to the extension of particular modes of governance, and whether or not specific regimes of governance are adequate in light of the circulation of particular bio-objects and the reach of bio-objectification processes. Here, it is instructive to follow particular bio-objects and to explore, when they become “upscaled,” for instance, to regulatory bodies of the European Union, and when bio-objects instead remain within a particular level of governance.

## Conclusion

When taken together, the dimensions outlined above form a multi-dimensional matrix of issues to consider when analyzing and comparing different modes of governance. They help to identify commonalities and differences across different countries, across bio-objects, and levels of governance. However, they also help to visualize temporal patterns or trends, such as an increasing trend for anticipatory modes of governance. Indeed, it seems that governance is decreasingly a practice that comes after the “bio-object;” instead, governance seems to be increasingly part of bio-objectification processes, seeking to enable, to “smooth,” and to shorten such processes. This suggests that the making of new life forms and the ordering of these life forms, but also of the processes that help engender them, are processes that are increasingly closer tied and intermingled.

## References

[1] Holmberg T, Schwennesen N, Webster A (2011). Bio-objects and the bio-objectification process.. Croat Med J.

[2] Metzler I, Webster A (2011). Bio-objects and their boundaries: governing matters at the intersection of society, politics, and science.. Croat Med J.

